# The Ubiquitin-Conjugating Enzyme Gene Family in Longan (*Dimocarpus longan* Lour.): Genome-Wide Identification and Gene Expression during Flower Induction and Abiotic Stress Responses

**DOI:** 10.3390/molecules23030662

**Published:** 2018-03-15

**Authors:** Dengwei Jue, Xuelian Sang, Liqin Liu, Bo Shu, Yicheng Wang, Jianghui Xie, Chengming Liu, Shengyou Shi

**Affiliations:** 1 Key Laboratory of Tropical Fruit Biology (Ministry of Agriculture), South Subtropical Crops Research Institute, Chinese Academy of Tropical Agricultural Sciences, Zhanjiang 524091, China; juedengwei@126.com (D.J.); anyue1220@126.com (X.S.); lolitallq@163.com (L.L.); bshbest@163.com (B.S.); ychw08@163.com (Y.W.); xiejianghui@21cn.com (J.X.); 2 College of Horticulture, South China Agricultural University, Guangzhou 510642, China; cmliu@scau.edu.cn

**Keywords:** longan, *UBC* gene, flower induction, abiotic stress, expression profiling

## Abstract

Ubiquitin-conjugating enzymes (E2s or UBC enzymes) play vital roles in plant development and combat various biotic and abiotic stresses. Longan (*Dimocarpus longan* Lour.) is an important fruit tree in the subtropical region of Southeast Asia and Australia; however the characteristics of the *UBC* gene family in longan remain unknown. In this study, 40 *D. longan* UBC genes (*DlUBCs*), which were classified into 15 groups, were identified in the longan genome. An RNA-seq based analysis showed that *DlUBCs* showed distinct expression in nine longan tissues. Genome-wide RNA-seq and qRT-PCR based gene expression analysis revealed that 11 *DlUBCs* were up- or down-regualted in the cultivar “Sijimi” (SJ), suggesting that these genes may be important for flower induction. Finally, qRT-PCR analysis showed that the mRNA levels of 13 *DlUBCs* under SA (salicylic acid) treatment, seven under methyl jasmonate (MeJA) treatment, 27 under heat treatment, and 16 under cold treatment were up- or down-regulated, respectively. These results indicated that the *DlUBCs* may play important roles in responses to abiotic stresses. Taken together, our results provide a comprehensive insight into the organization, phylogeny, and expression patterns of the longan UBC genes, and therefore contribute to the greater understanding of their biological roles in longan.

## 1. Introduction

Ubiquitylation (also called ubiquitinylation or ubiquitination) is the covalent attachment of ubiqutin (Ub) to substrate proteins. Ubiquitin is a small protein containing 76 amino acids that is highly conserved in eukaryotes; only three residues differ between yeast, human, and plant species [1,2]. The process of protein ubiquitination (ubiquitin-proteasome system, UPS) is mediated through the action of three enzymes, E1 (Ub-activating enzyme, UBA), E2 (Ub-conjugating enzyme, UBC), and E3 (Ub ligase) [3]. Ub is first linked to E1 through an ATP-dependent reaction that creates a thioester bond between the C-terminus of Ub and the cysteine in the active site of E1. The activated Ub is then transferred via a thioester bond from E1 to a cysteine residue of E2, before ubiquitin is finally transferred either to a substrate directly aided by E3 or to a cysteine of an alternative ubiquitin protein ligase (E3s) by a second transthiolation reaction to the target substrate. Finally, the target proteins are degraded by the 26S proteasome. In the ubiquitylation system, substrate specificity is mainly determined by E2 and E3 enzymes.

The E2 enzymes contain a conserved catalytic domain called the UBC domain, which comprises approximately 140–200 amino acids [4]. In addition to the core E2 domain, many detected isoforms contain various N- and C-terminal extensions that are proposed to influence target recognition and localization [5]. Based on the UBC domain and the N- and C-terminal extensions, E2 proteins are divided into four classes. Class I E2s only contain the UBC domain; class II E2s contain the UBC domain and the N-terminal extensions; class III E2s contain the UBC domain and the C-terminal extensions; and class IV E2s contain the UBC domain and both N- and C-terminal extensions [4,6]. The UBC protein family is very different from the ancestral eukaryotes that possess fewer members (e.g., ≤20 in algae), to the multicellular plants and animals that possess more diverse proteins (e.g., ≥70 in banana and maize) [7,8]. In addition, the ubiquitin-like conjugating enzymes (UBLs), which include the related ubiquitin- (RUB) conjugating enzymes and the SUMO-conjugating enzymes, as well as the ubiquitin-conjugating enzyme variants (UEVs), were also found among the E2 classes. For example, a total of 48 UBC domain-containing proteins have been identified in *Arabidopsis* [9], of which three have thioester-linked UBLs, two are RUB-conjugating enzymes (RCE1, At4g36800 and RCE2, At2g18600), and one is a SUMO-conjugating enzyme (AtSCE1, At3g57870). Eight other UBC proteins lack the Cys active site and are referred to as ubiquitin-conjugating enzyme variants (UEVs) that are not active by themselves, leaving 37 potential E2s [10].

Plant UBC proteins play an important role in regulating plant growth, development, and many abiotic stress reactions. For example, the *Arabidopsis* ubiquitin-conjugating gene *AtUBC13* has been implicated in epidermal cell differentiation and iron deficiency responses [11,12]. AtUBC32 is an ERAD (endoplasmic reticulum-associated protein degradation) component that functions in brassinosteroid-mediated salt stress tolerance [13]. AtUBC22 is required for female gametophyte development and is likely to be involved in Lys11-linked ubiquitination [14]. A tomato UBC13-type homologous protein, FNI3, is involved in the regulation of Fen-mediated immunity [15], and the tomato-specific E2 regulates fruit ripening [16]. The virus induced gene silencing of a *Triticum aestivum* ubiquitin-conjugating enzyme 4 (TaU4) gene expressed in wheat leaves results in delayed development of disease symptoms, and reduced *Septoria* growth and reproduction [17].

Flowering is a crucial developmental process in the life cycle of plants [18]. The molecular and genetic basis of flowering is well studied in the model plant *Arabidopsis* [19,20,21]. In *Arabidopsis*, five major flowering pathways have been identified, including the photoperiod pathway, the vernalization pathway, the autonomous pathway, the GA pathway, and the aging pathway [22]. These pathways mediate responses to various stimuli, such as light, age, circadian clock, photoperiod, temperature, abiotic stresses, and hormones. Several genes such as *CONSTANS* (*CO*), *FLOWERING LOCUS T* (*FT*), *SUPPRESSOR OF OVEREXPRESSION OF CO1* (*SOC1*), and *FLOWERING LOCUS C* (*FLC*) are key components in flowering signal pathways [23]. In addition, a number of transcription factor (TF) family genes, such as MADS-domain TFs [24], NACs [25], MYBs [26], DREBs [27], WRKYs [28] and ASMTs [29] are involved in flowering regulation. Ubiqitination appears to play a role in most phases of floral development [11]. The RING-finger E3 ligase CONSTITUTIVE PHOTOMORPHOGENIC1 (COP1) is a central regulator of light-dependent physiological processes such as photomorphogenesis, circadian oscillation, and floral transition, and is also involved in ambient temperature-dependent flowering [30,31,32]. The light/dark-dependent localization of COP1 is regulated by E2 COP10 through non-K48-linked polyubiquitin, as well as by derubylation by CSN, which is emerging as a regulator of SCF E3 ligase activity [33]. In *Arabidopsis*, AtUBC1/AtUBC2 act together with the E3s HUB1/HUB2 in H2B ubiquitylation, which is involved in the activation of floral repressor genes [34]. However, little is known about the function of E2s in flower induction, especially in fruit trees.

Longan (*Dimocarpus longan* Lour.) is an important subtropical fruit tree belonging to the family Sapindaceae, which is grown in many subtropical and tropical countries, with the majority of its production being in Southeast Asia and Australia [35]. Common longan varieties, such as “SX”, one of the main varieties in China, exhibit the “seasonal flowering” (SF) habit; floral bud induction requires a period of low temperature, and only the terminal meristem differentiates into an inflorescence. In order to obtain a stable high yield, off-season flowering in longan is achieved by chemical treatment with potassium chlorate (KClO_3_) [36,37]. However, the induction effect varies in different regions and tree varieties. Therefore, the difficulty in flowering of these trees is a considerable problem in the longan industry. Therefore, the study of the molecular regulatory mechanisms of flower induction in longan is particularly important for understanding and solving the problems associated with flowering. One cultivar of longan, “SJ”, flowers and bears fruits throughout the year under both high and low temperature, exhibit the “perpetual flowering” (PF) habit. This cultivar does not require a controlled environment; hence, it is a good model for studying longan flowering.

In the present study, we performed a genome-wide identification of UBC proteins in longan and analyzed their gene structures, conserved motifs, *cis*-elements and expression patterns in nine different tissues. This study also determined the expression profiles of longan UBC genes (*DlUBC*) during the three flowering stages in two longan cultivars, and measured their transcript abundance in response to different phytohormone treatments and abiotic stresses. This study provides a basis for future studies on the evolution and functions of the *DlUBC* gene family.

## 2. Results

### 2.1. Identification and Characterization of UBC Family Genes in Longan

**Figure 1:**
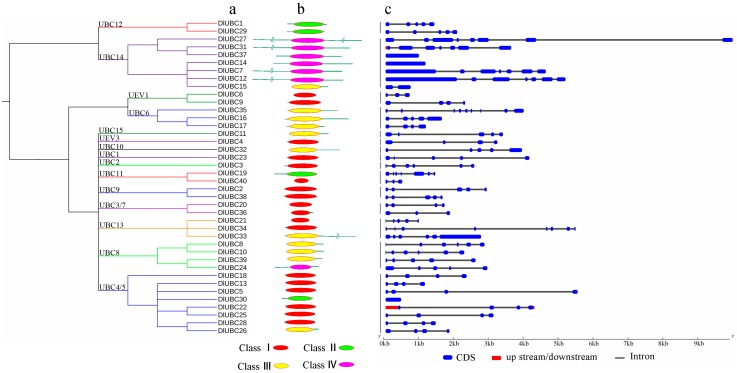
Phylogenetic relationships, architecture of conserved protein motifs and gene structure in *DlUBC* genes from longan. (**a**) The neighbor-joining (NJ) tree on the left includes 40 *DlUBC* proteins from longan; (**b**) The architecture of conserved protein motifs of *DlUBC* proteins with the name of each corresponding protein is shown on the left. The position of the UBC domain is indicated in the panel. The different colors indicate the four E2 subtypes of the UBC domains; (**c**) Exon/intron structures of UBC genes from longan. Untranslated 5′ and 3′ regions, exon(s), and intron(s) are represented by red, blue boxes, and gray lines, respectively. The scale bar represents 1000 bp.

In total, 72 putative longan UBCs were identified in the longan genome database using HMM and BLASTp search methods (Table S2). On the basis of the UBC domain scanning and sequence alignment, 24 genes without a complete predicted UBC domain and 8 redundant genes (*Dlo_012456.1*, *Dlo_015113.1*, *dlo_034620.1*, *dlo_034680.1*, *dlo_034691.1*, *Dlo_018814.1*, *Dlo_023089.1* and *Dlo_008132.1*) were removed (Table S2). Finally, 40 potential UBC proteins were identified in this study. We named these 40 UBC genes *DlUBC1* to *DlUBC40* based on their chromosomal locations. Subsequently, gene characteristics, including the length of full-length sequence, ORF, protein sequence, molecular weight (MW), and isoelectric point (pI) were analyzed (Table 1 and Figure 1). The full-length sequences ranged from 446 bp (*DlUBC30*) to 9890 bp (*DlUBC27*), with an average of 2644 bp. The length of the ORFs ranged from 243 bp (*DlUBC40*) to 3438 bp (*DlUBC12*), averaging 755 bp. The length of the protein sequences ranged from 80 amino acids (*DlUBC40*) to 1145 amino acids (*DlUBC12*), averaging 251 amino acids. The protein MW ranged from 9.04 kDa (*DlUBC40*) to 126.46 kDa (*DlUBC12*), averaging 28.13 kDa. The pI ranged from 4.12 (*DlUBC12*) to 9.82 (*DlUBC14*), with an average of 6.71.

### 2.2. The Gene Structure and Motif Composition of the Longan UBC Gene Family

To further understand the similarity and diversity of motif composition among different *DlUBCs*, a neighbor-joining (NJ) phylogenetic tree was constructed using all full-length UBC protein sequences from longan. Using the yeast and *Arabidopsis* UBC proteins as references for classification, we subdivided the 40 UBC members from longan into 15 groups according to sequence similarity and topology (Figure 1a).

According to the presence of the UBC domain and the N- or C-terminal extensions that are typically responsible for the functional differences between E2s, the E2s are divided into four types [4]. In the present study, 18 *DlUBC* proteins belong to Class I E2s; four, eleven and seven *DlUBC* proteins belong to Classes II, III, and IV E2s, respectively (Figure 1b). The exon/intron structure analysis for the 40 longan UBC genes indicated that most of the coding sequences were disrupted by introns, except for the three genes *DlUBC14*, *DlUBC30* and *DlUBC37* (Figure 1c). The number of introns in the *DlUBC* genes ranged from zero to eight, with approximately 55% of the *DlUBC* genes possessing three or four introns. Phylogenetic analysis of *DlUBC* genes showed that most of the genes that clustered into the same group exhibited similar exon/intron structures. For example, all the members of groups UBC9 and UBC12 contained four introns (Figure 1c).

The longan UBC protein sequences were subjected to MEME (Multiple EM for Motif Elicitation); a total of 15 distinct motifs were identified and were designated as motif 1 to 15. The details of the conserved amino acid sequences and their lengths are shown in Table S3. The most common motif at the N-terminal was motif 4 (HPNIYSNGSICLDIL), which was found in 34 out of 40 (85%) longan UBCs, and motif 1 was also common at the N-terminal (77.5%). Most members in the same group shared similar motifs, and high variance was observed between the different groups (Figure S1). The results also showed that some motifs were only found in one or two groups of *DlUBC* proteins. For example, motif 12 and 14 were found exclusively in groups UBC14 and UBC17, and motif 11 was only present in group UBC 4/5.

### 2.3. Phylogenetic Analysis of the DlUBC Genes

**Figure 2:**
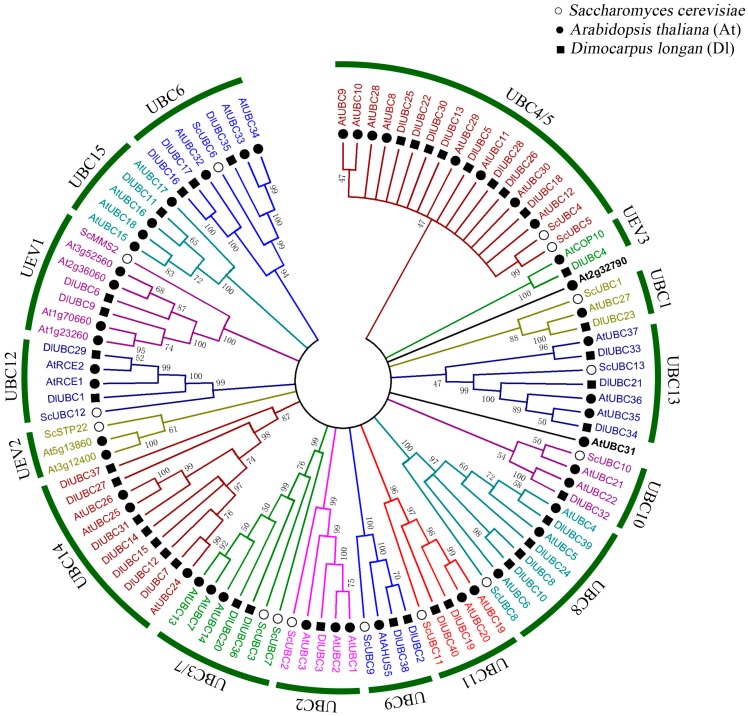
Phylogenetic analysis of the longan UBC proteins with orthologous members from *Saccharomyces cerevisiae* and Arabidopsis. The neighbor-joining (NJ) phylogenetic tree was constructed with MEGA 6.0 software. Different groups of *DlUBC* proteins are indicated by a circle and different colors.

To categorize and investigate the evolutionary relationships of *DlUBC* genes, we constructed a phylogenetic tree by aligning the full-length UBC protein sequences for members of *Saccharomyces cerevisiae* (15), *A. thaliana* (48), and *D. longan* (40) (Figure 2). As shown in Figure 2, the results of our phylogenetic analysis revealed that all of the 103 UBC proteins could be categorized into 15 groups, and one group which doesn’t contain any *DlUBC* based on >46% bootstrap support. The groups UBC9 and UBC12 functioned in SUMO and RUB1 conjugation pathways, respectively, and three UEV groups which lack the Cys active site. These groups were designated as UBC1, UBC2, UBC3/7, UBC4/5, UBC6, UBC8, UBC9, UBC10, UBC11, UBC12, UBC13, UBC14, UBC15, UEV1, UEV2 and UEV3. The 40 UBC members of longan were further divided into 15 groups that only contained two UEV group (UEV1 and UEV3). Interestingly, the groups UBC14, UBC15 and UEV3 were absent from the yeast genome, indicating that these groups may be plant-specific or were lost from the most common shared ancestor of yeast and plants. Additionally, UBC4 and UBC5 and UBC3 and UBC7, shared high homology; therefore, these groups were clustered as groups UBC4/5 and UBC3/7 in our study.

### 2.4. Tissue-Specific Expression Patterns of DlUBC in Longan

**Figure 3:**
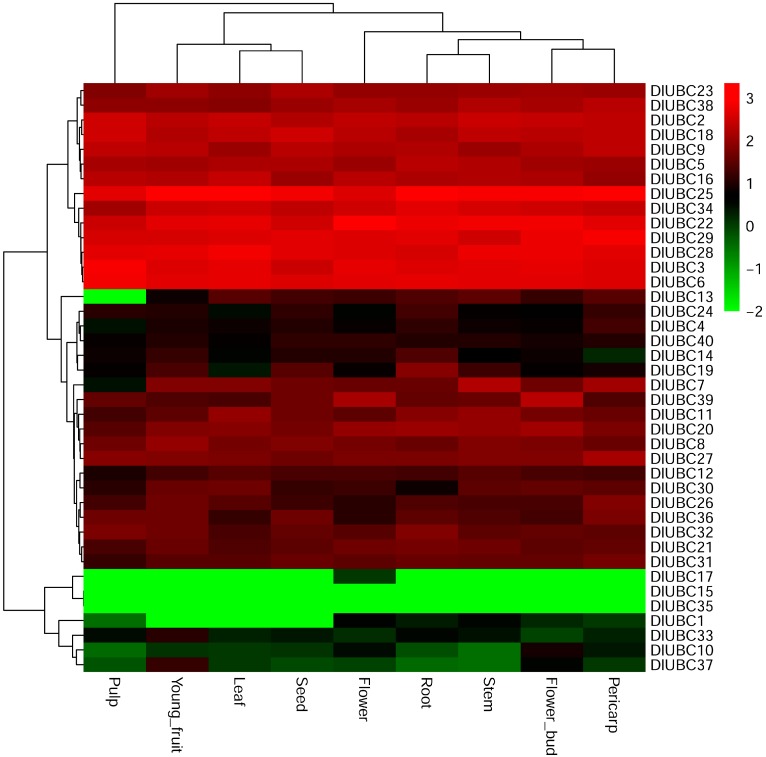
Expression profiles of *DlUBC* genes across different tissues and developmental stages in longan. Heat map of the hierarchical clustering of 40 *UBC* genes across different tissues analyzed in this study. The color scale represents log_10_ expression values; red and green colors indicate lower or higher transcript abundance compared to the relevant control, respectively.

To assess the potential functions of *DlUBC* genes during longan development, the expression profiles of 40 *DlUBC* genes in root, stem, leaf, seed, young fruit, pulp, pericarp, flower, and flower bud were investigated by RNA-seq analysis. The RNA-seq data for 40 *DlUBC* genes (Table S4) was downloaded from the NCBI database and a heat map of their expression was generated (Figure 3). Results showed that almost all *DlUBCs* were expressed in flowers and flower bud except *DlUBC15* and *DlUBC35*. Furthermore, 92.5% of the *DlUBCs* were expressed in the pericarp, root, stem and young fruit; and 90% were expressed in the leaf, pulp and seed. Approximately 87.5% (35 of 40) of the *DlUBC* genes were expressed in each tested tissue. A total of three *DlUBC* genes (*DlUBC10*, *33*, and *37*) had low expression in all tested tissues. Furthermore, *DlUBC15* and *DlUBC35* could not be detected in all tested longan tissues and *DlUBC17* only displayed a significantly low expression in flowers. The *DlUBC1* gene showed no expression in leaf, seed, and young fruit, and low expression in the remaining tissues (Table S4). It is worth noting that 14 genes (*DlUBC2*, *3*, *5*, *6*, *9*, *16*, *18*, *22*, *23*, *25*, *28*, *29*, *34* and 38) were highly expressed in the nine longan tissues.

### 2.5. Comparative Expression Profiles of the Two Longan Species during Flowering

**Figure 4:**
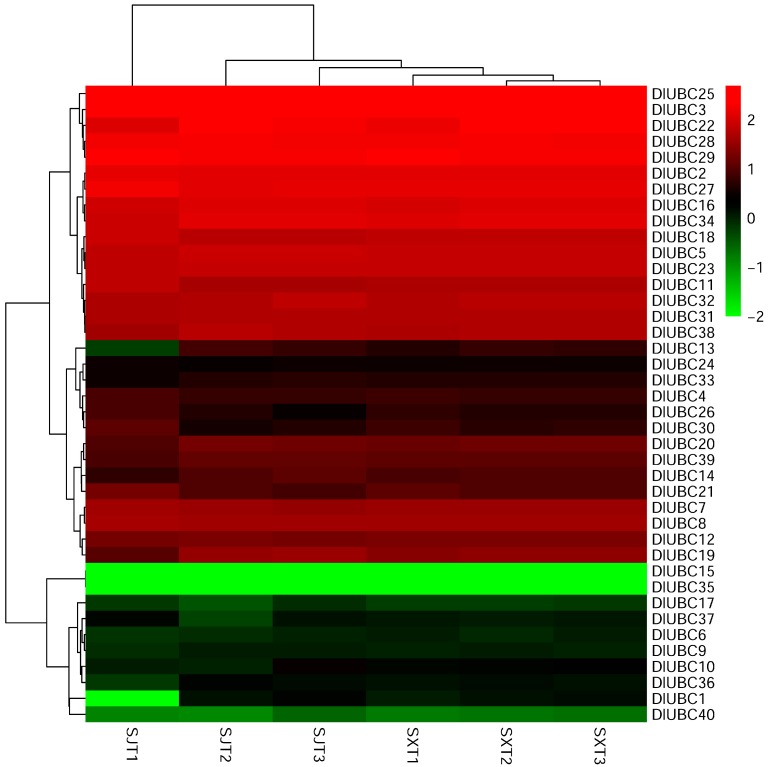
Heat map of the comparative expression level of *UBC* genes during the three flowering stages of the ‘SJ’ and “SX”. Genes with comparatively low expression values are shown using shades of green, and high expression values are represented using shades of red. The three flowering stages of SJ are indicated by SJT1, SJT2 and SJT3. The three flowering stages of SX are indicated by SXT1, SXT2 and SXT3.

Flowering is a critical event in the life cycle of plants, especially in fruit trees. However, the mechanisms of flower induction in longan have not been elucidated. In the present study, we also analyzed the expression patterns of 40 *DlUBC* genes in two longan species during the three flowering stages by RNA-seq analysis (Table S5). One heat map was constructed based on the log_10_ (FPKM + 0.01) values for the 40 *DlUBC* genes (Figure 4). *DlUBC* genes differentially expressed during the three flowering stages of the two longan species were identified based on the criteria for *p* values < 0.05 and fold changes ≥ 2. Results showed that all the 40 *DlUBC* genes were constitutively expressed during the three flowering stages in the cultivar ‘SX’, while 11 *DlUBC* genes were differentially expressed in ‘SJ’. Among these 11 *DlUBC* genes, seven (*DlUBC1*, *10*, *13*, *14*, *19*, *20* and *36*) were up-regulated during the three flowering stages, and three genes (*DlUBC21*, *26* and *30*) were down-regulated. Moreover, one gene (*DlUBC37*) was down-regulated in the first two stages and then upregulated in the third stage.

**Figure 5:**
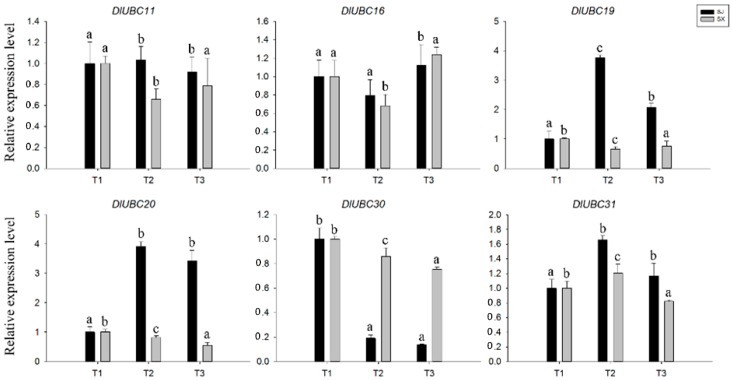
Relative expression levels of six *DlUBCs* during the three flowering stages of the two longan species by qRT-PCR. For each gene, the relative expression level in T1 (dormant apical bud) was set as one, and the longan *actin* gene was used as the internal expression control. The data represents the mean ± SD of three replicates. Values with the same letter were not significantly different when assessed using Duncan’s multiple range test (*p* < 0.05, *n* = 3).

To validate the expression levels obtained from the RNA-seq data, six *DlUBC* genes (*DlUBC11*, *16*, *19*, *20*, *30* and *31*) were randomly selected from six different longan UBC groups for quantitative real-time reverse transcription polymerase chain reaction (qRT-PCR) analysis (Figure 5). The transcript levels of all six *DlUBC* genes did not exhibit any significant differences in ‘SX’ longan between the three flowering stages. In addition, the relative expression level of *DlUBC11*, *DlUBC16* and *DlUBC31* did not exhibit any significant differences in ‘SJ’ during the three flowering stages. The expression levels of *DlUBC19* and *DlUBC20* were upregulated at the second and third stage. The transcript levels of *DlUBC30* were downregulated at the second and third stage. In general, the expression levels obtained by qRT-PCR for these genes are similar to the results obtained from the RNA-seq data.

### 2.6. Differential Regulation of DlUBCs in Response to Stress and Hormonal Treatments

**Figure 6:**
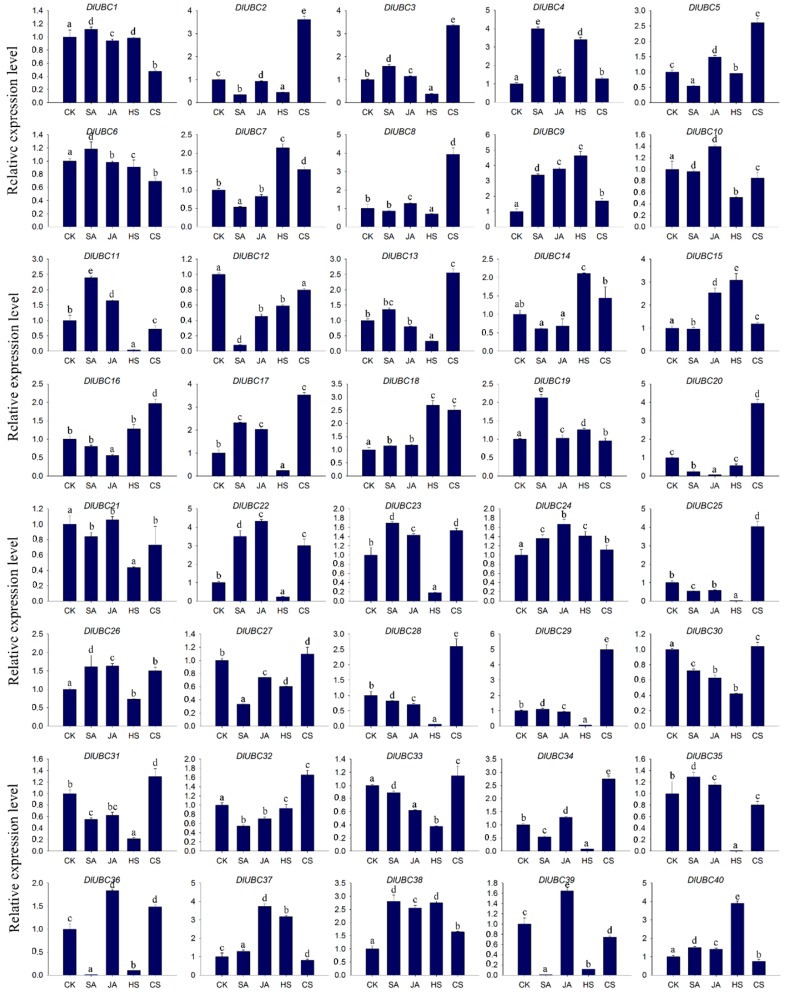
Expression patterns of *DlUBC* genes under various hormonal and abiotic stresses. The *x*-axis indicates various treatments and the *y*-axis indicates the relative gene expression level. Error bars indicating SD were obtained from three independent biological replicates. Values with the same letter were not significantly different when assessed using Duncan’s multiple range test (*p* < 0.05, *n* = 3). SA = salicylic acid, JA = jasmonic acid, HS = heat stress, and CS = cold stress.

Subsequently, the expression patterns of 40 *DlUBC* genes were investigated in response to hormonal and various stress using qRT-PCR (Figure 6). 

In the 40 *DlUBC* genes, *DlUBC6*, *10*, *16*, *24*, *26* and *32* showed no significant differential expression in response to the treatments. The remaining 34 *DlUBC* genes were up-regulated or down-regulated in at least one tested treatment. We identified 17 *DlUBC* genes with different expression levels under SA treatment, in which seven genes (*DlUBC4*, *9*, *11*, *17*, *19*, *22* and *38*) were up-regulated and 10 genes (*DlUBC2*, *5*, *12*, *20*, *25*, *27*, *32*, *34*, *36* and *39*) were down-regulated. Meanwhile, five genes (*DlUBC9*, *15*, *22*, *37* and *38*) were induced, while two genes (*DlUBC20* and *DlUBC21*) were inhibited by MeJA treatment. After heat treatment, 17 (*DlUBC2*, *3*, *11*, *13*, *17*, *22*, *23*, *25*, *28*, *29*, *30*, *31*, *33*, *34*, *35*, *36* and *39*) and nine (*DlUBC4*, *7*, *9*, *14*, *15*, *18*, *37*, *38* and *40*) genes were down-regulated or up-regulated, respectively. A total of 13 *DlUBC* genes (*DlUBC2*, *3*, *5*, *8*, *13*, *17*, *18*, *20*, *22*, *25*, *28*, *29*, and *34*) were up-regulated, and only one gene (*DlUBC1*) was down-regulated under cold treatment.

### 2.7. Analysis Related Cis-Elements in the Candidate DlUBC Genes

**Figure 7:**
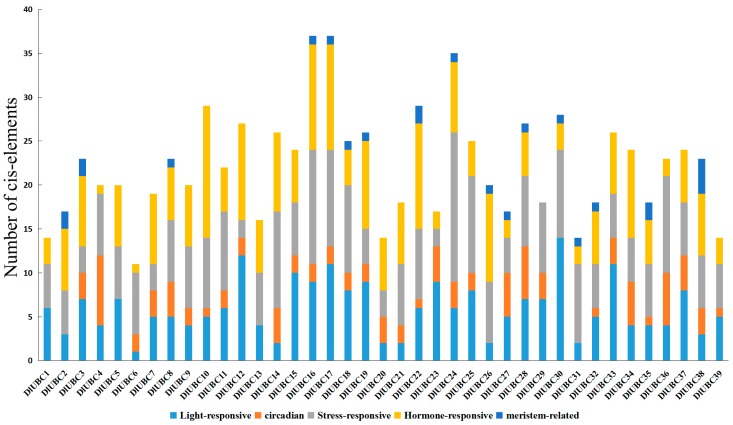
Predicted *cis*-elements in the promoter of the *DlUBC* genes. The 1.5 kb sequences of 40 *DlUBC* genes were analyzed with the PlantCARE software.

To further analyze the potential roles of *DlUBC* genes in response to various responses, a 1.5 kb upstream regulatory region (promoter) of *DlUBC* genes were used to search for *cis*-elements. Of the 40 genes, 1.5 kb upstream regulatory region could be fetched in 39. Only six promoter bases of *DlUBC40* could be fetched as only these many bases are available upstream in the assembled scaffold which it belongs. All of *DlUBC* genes shared the light-responsive boxes and stress-responsive boxes in their promoter. Hormones-related *cis*-elements, such as MeJA, salicylic acid, gibberellin, auxin and ethylene, were existed in the promoter of all *DlUBC* genes except *DlUBC29*. Additionally, circadian-related *cis*-elements were found in the promoter of thirty-two *DlUBC* genes and Meristem-related *cis*-elements only presented in the promoter of seventeen *DlUBC* genes (Figure 7, Tables S6 and S7). These results indicated that *DlUBC* genes may be regulated by various *cis*-elements within the promoter during growth and stress responsive.

## 3. Discussion

Ubiquitin-conjugating enzymes (E2s) have been characterized and analyzed in both prokaryotes and eukaryotes [9]. However, neither a genome-wide identification nor a comprehensive assessment of this gene family in longan has been previously reported. Recently, the successful sequencing of the longan genome has made it possible to analyze this gene family at the whole-genome level [38]. 

In the present study, a total of 40 UBC genes were identified in the longan genome (472 Mb), of which two are RUB-conjugating enzymes (*DlUBC1* and *DlUBC29*), and two are SUMO-conjugating enzymes (*DlUBC2* and *DlUBC38*). In addition, three UEV genes (*DlUBC4*, *DlUBC6* and *DlUBC9*) were also found in the longan UBC family (Figure 2). This result is consistent with the findings in other species. For example, there are one RUB-conjugating enzyme (ScUBC9), one SUMO-conjugating enzyme (ScUBC12), and two UEV proteins (ScMMS2, NP_011428 and ScSTP22, NP_009919) among the 15 *S. cerevisiae* UBC proteins [9]. In *Arabidopsis*, there are two AtUBC RUB-conjugating enzymes, a SUMO-conjugating enzyme, and eight AtUBCs that are UEVs [39]. In rice, there are three RUB-conjugating enzymes (OsUBC1, 2, and 3), three SUMO-conjugating enzymes (OsUBC4, 5, and 6), and four UEVs (OsUBC28, 29, 30 and 31) [40]. The genome sizes of *Arabidopsis*, rice, tomato, maize, papaya, and longan are ~125, ~466, ~900, ~2300, ~371 and ~472 Mb, respectively. However, the number of UBC genes in *Arabidopsis*, rice, tomato, maize, papaya, and longan is 48, 48, 52, 75, 34 and 36, and not correlated with the genome size [8,16,39,40,41]. Several studies have shown that segmental duplications are largely responsible for the expansion of gene families in other plants, such as maize, pineapple and soybean [8,42,43]. Unfortunately, there is a lack of information on the chromosomes of longan, therefore we were unable to perform segmental and tandem duplication analysis of *DlUBCs*.

The phylogenetic relationship analysis showed that all the 103 UBC proteins could be categorized into 16 groups. Eleven groups (including UBC1, 2, 3/7, 4/5, 6, 8, 9, 10, 11, 12 and 13) were present in *S. cerevisiae*, *A. thaliana*, and *D. longan*, as well as in rice, tomato, and maize [8,9,16,39,40]. This result suggests that these 11 groups may have evolved before the divergence of the ancestor of yeast and plants. The ubiquitin E2 enzyme variant (UEV) proteins are similar to E2s in both sequence and structure, but lack a catalytic cysteine residue, and thus are unable to form a thiol-ester linkage with ubiquitin [44]. To comprehensively understand the function of longan UBC proteins, the UEVs were also considered. In the present study, three UEV genes (*DlUBC4*, *DlUBC6* and *DlUBC9*) were existed in the longan genome, which is fewer than in *Arabidopsis*, rice, and maize [8,39,40]. Additionally, the groups UBC14 and UBC15 are absent in the yeast genome, indicating that these groups may have been lost in the ancestor of yeast or have evolved after the divergence of the ancestor of yeast and plants. The number of UBC genes differed among the groups too. For instance, the UBC1, 2, 10 and 15 groups only contained one *DlUBC* gene each, while the largest group (UBC4/5) included eight genes. Similar results were found in other studies [7,8,41], suggesting group UBC4/5 might have more diverse functions than other groups. In addition, there are some minor differences in the topologies of the *UBC* genes in *Arabidopsis* among different studies. For example, *AtUBC31*, clustered into the UBC 4/5 group in the previous studies [7,8], was not placed in any groups in our study. These differences in protein classification could have resulted from different parameter settings or methods during the phylogenetic analyses.

Accumulated data suggests that UBC genes play important roles in diverse plant development processes and have different expression patterns in different organs [8,45]. For example, in *Arabidopsis*, *AtUBC1* and *AtUBC2* are ubiquitously expressed in roots, leaves, flowers, and seedlings [34]. The double mutant of *Arabidopsis UBC13A/B* displays strong phenotypes, including shortened primary roots, a reduced number of lateral roots, and few and short root hairs [46,47]. In banana, *MaUBC10*, *11*, *33*, *34*, and *61* are highly expressed in most organs, but especially in roots, stems, and leaves; while *MaUBC6*, *11*, *34*, *35*, *45*, and *61* were highly expressed in stems, implying that these genes were likely to be involved in basal metabolic or housekeeping functions in the banana development [7]. In papaya, all 34 *CpUBC* genes showed organ-specific expression patterns; nineteen (*CpUBC1*, *2*, *3*, *5*, *6*, *9*, *10*, *11*, *12*, *15*, *17*, *20*, *23*, *24*, *26*, *30*, *31*, *33* and *34*) were highly expressed in male flowers and two genes (*CpUBC21* and *CpUBC22*) were expressed in female flowers which suggests that these genes may be involved in the development of floral sex organs [41]. Consistent with the previous studies, in the present study, most *DlUBC* genes were expressed widely in the different organs that we examined, suggesting that *DlUBC* genes may be play diverse roles in longan organ development (Table S4 and Figure 3). For example, *DlUBC3*, belonging to group UBC2, is orthologous to *AtUBC1* and *AtUBC2*, and has ubiquitous expression in roots, leaves, and flowers. Meanwhile, our results also showed that several genes showed a specific expression in longan organs. For instance, *DlUBC17* was only weakly detected in flowers and *DlUBC19* were higher expressed in roots, which indicated that these two genes might be involved in the development of flowers or roots, respectively. In general, these results indicate that *DlUBC* genes may play various roles in the development of different longan tissues.

Flowering is a transition from vegetative to reproductive development, and is one of the most important events in the life cycle of higher plants, because it is vital for reproductive success [18,48]. This transition is coordinated through a diverse array of signaling networks that integrate various endogenous and exogenous signals [23]. In past decades, we have gained increasing knowledge of flowering time regulation in model species such as *Arabidopsis* [20] and many family genes involved in this regulation have been identified, such as *WRKYs* and *ASMT* [29,49]. Although UBC proteins have important roles in plant growth and development, little is known about its functions in the process of flower induction. For example, the *Arabidopsis* UBC1 and UBC2, together with two closely related RING-type E3s called HUB1 (HISTONE MONOUBIQUITINATION1) and HUB2, are involved in histone 2B monoubiquitination and the regulation of flowering time [34,50]. For longan, several studies indicated that the homologues, such as *SHORT VEGETATIVE PHASE* (*SVP*), *GIGANTEA* (*GI*), *F-BOX 1* (*FKF1*), *EARLY FLOWERING 4* (*ELF4*), *CO* and *FLC*, might be involved in the control of flowering by using RNA-seq analysis [36,37]. However, to date, the role of UBC proteins in the flower induction in longan has not been previously studied. In the present study, the expression of 40 *DlUBC* genes was evaluated during three different flowering stages by using RNA-seq. Interestingly, the results showed that all of the 40 *DlUBC* genes were constitutively expressed in the three flowering stages in the “SX” longan variety, which flowers only once a year. Additionally, 11 *DlUBC* genes were differentially expressed in the “SJ” longan variety, which flowers throughout the year (Figure 4). Meanwhile, the expression levels measured by qRT-PCR for Six *DlUBC* genes (*DlUBC11*, *16*, *19*, *20*, *30* and *31*) randomly selected were similar to the results obtained from the RNA-seq data (Figure 5). These results suggesting that those *DlUBC* genes may participate in flower induction, especially involved in the regulation of PF habit in longan. However, *DlUBC3*, which is orthologous to *AtUBC1* and *AtUBC2*, did not show any change during the three flowering stages. This result is consistent with the expression of *AtUBC3*, the other member of group UBC 2, which does not show redundancy with *AtUBC1* and *AtUBC2*. Furthermore, only the *UBC1 UBC2* double mutant without *UBCs* has an early flowering phenotype [50]. We speculate that these orthologous genes may be involved in different signaling pathway in *Arabidopsis* and longan. In summary, we propose that these 11 *DlUBC* genes play crucial roles in longan flowering, and need further investigation.

Longan is frequently challenged by abiotic stressors such as high salinity, drought, and extreme temperatures. Recent studied have shown that UBC proteins are widely involved in signaling and response to these stresses in many species [45]. For example, three rice genes (*OsUBC2*, *5* and *18*) and five *Arabidopsis* genes (*AtUBC13*, *17*, *20*, *26*, and *31*) in the UBC family were significantly down-regulated, whereas only three rice genes (*OsUBC13*, *15* and *45*) were significantly up-regulated under salt and drought stresses [51]. In maize, 16, 20, and over half of the *ZmUBC* genes (48 genes) were significantly up-regulated under drought, cold, and salt conditions, respectively [8]. Consistent with previous studies, in the present study, the mRNA levels of 26 and 14 *DlUBC* genes were up- or down-regulated by heat or cold treatment, respectively (Figure 6). These results suggest that those genes might play important roles under high or low temperature conditions. To date, several studies indicated that E3 proteins respond to hormonal treatment. For instance, the RING E3 ligases AIRP1 and AIRP2 are responsible for reducing root growth rate in response to ABA [52]. However, there are few studies on the interaction between UBC protein and hormones. In the present study, 17 and seven *DlUBC* genes had different expression levels during SA and MeJA treatments, respectively. These results indicate that these *DlUBCs* could potentially play vital roles in stress and hormone responses.

Differential responses of some family genes are regulated by the presence of *cis*-elements in their promoter region [53,54,55]. For example, *Morus013217* which contained three LTREs in its promoter regions showed a strong response to cold stress [53]. Similar results also found in our study. For instance, one HSE *cis*-element was found in the promoter regions of *DlUBC9*, which showed an induce response to heat stress. *DlUBC9* and *DlUBC22* showed responsiveness to SA treatment, and TCA-elements were found in their promoters (Figure 7 and Table S7). Thus, these *cis*-elements could provide more evidence of *DlUBC* genes in response to different stress or hormonal signaling.

## 4. Materials and Methods

### 4.1. Identification of the Longan Conjugating Enzyme Family Gene

Genome sequences of longan have recently become available and were downloaded from the NCBI Sequence Read Archive (SRA315202) or ftp://climb.genomics.cn/pub/10.5524/100001_101000/100276/ [38]. To identify potential members of the *DlUBC* gene family, the hidden Markov model (HMM) profile of the ubiquitin-conjugating enzyme domain (PF00179) was extracted from the Pfam database (http://pfam.xfam.org/family/PF00179) [56] and used to search for putative UBC proteins from the longan genome sequence with HMMER 3.0 (http://hmmer.janelia.org/). The default parameters were adopted, and the cutoff value was set to 0.01. Subsequently, BLAST searches using all *Arabidopsis* and *Saccharomyces* UBC protein sequences as queries were performed with default parameters. Finally, all candidate sequences were examined to confirm the presence of the conserved UBC domain (PF00179) using SMART (http://smart.emblheidelberg.de) and Pfam (http://pfam.xfam.org) database analyses [57].

### 4.2. Sequence Analysis

The molecular weight (MW), number of amino acids, open reading frame (ORF) length, and isoelectric point (pI) of *DlUBCs* were calculated using ExPASy online tools (http://expasy.org/tools/) [58]. Gene Structure Display Server (GSDS) version 2.0 was used to display the intron and exon junctions and the arrangements of *DlUBC* genes [59]. The conserved motifs of *DlUBC* proteins were identified by MEME (http://meme.sdsc.edu/meme/cgi-bin/meme.cgi) [60] with the following optimized parameters: any number of repetitions, a maximum number of 15 motifs, and an optimum width of each motif between six and 50 residues.

### 4.3. Sequence Alignment, Cis-Elements in the Promoters and Phylogenetic Analysis

Sequences of 15 *S. cerevisiae* and 48 *Arabidopsis* UBC proteins were described previously [9,39] and obtained from the *Saccharomyces* Genome Database (http://www.yeastgenome.org/) and TAIR (http://www.arabidopsis.org/), respectively. The 1,500-bp sequences upstream of the transcription start site of candidate *DlUBC* genes were extracted from the longan genome sequences. PlantCARE software (http://bioinformatics.psb.ugent.be/webtools/plantcare/html/) was used for searching the *cis*-acting elements [61]. For phylogenetic analysis, the UBC protein sequences of *S. cerevisiae*, *Arabidopsis*, and longan were aligned using Clustal X 1.83 (http://www.bio-soft.net/fomat.html). Based on this alignment, a bootstrapped neighbor-joining (NJ) tree was constructed using MEGA version 6.0 (http://www.megasoftware.net) and a bootstrap test replicated 1000 times [62]. To assess the phylogenetic relationships among the members of the longan *UBC* gene family, a phylogenetic tree was constructed according to the alignment of only longan proteins. All *DlUBC* proteins were classified into groups based on their structural features and evolutionary relationships.

### 4.4. Expression Analysis of Longan UBCs in Various Tissues and at Different Flowering Stages

The RNA-seq data for the “SJ” variety was downloaded from the NCBI Sequence Read Archive (GSE84467) and used to analyze the expression patterns of *UBC* genes in the root, stem, leaf, seed, young fruit, pulp, pericarp, flower, and flower buds. Fragments per kilobase of exon model per million mapped values were log_10_-transformed, and heat maps with hierarchical clustering were designed using the software Mev 4.9.0 (http://tm4.org) [63].

Three pairs of nine-year-old “SJ” trees, which exhibit the perpetual flowering habit, and “SX” trees, which exhibit the seasonal flowering habit, were used in this study. Those trees were grown at experimental orchard of the South Subtropical Crops Research Institute of the Chinese Academy of Tropical Agricultural Science in Zhanjiang (110°16′ E, 21°10′ N), China. Three different kind apical buds from the dormant stage (before the emergence of floral primordial) (T1), the emergence of floral primordia stage (T2), and the floral organ formation stage (T3) of “SJ” and “SX” were identified by a histological analysis [64]. Samples of each stage of “SJ” are abbreviated SJT1, SJT2, SJT3, and samples of different development stages in “SX” are abbreviated SXT1, SXT2 and SXT3. The samples obtained for the SXT1, SXT2 and SXT3 were collected on 20 November 2016, 24 December 2016, and 1 January 2017, respectively. The three kind samples of “SJ” were obtained at the same time compared to “SX”. For each sample, we used three biological replicates from three different trees. Each biological replicate contained mixed buds. All samples were collected from 10:00 to 12:00 a.m., and were frozen immediately in liquid nitrogen and stored at −80 °C. Total RNA were extracted separately from the bud samples of three biological replicates using the quick RNA Isolation Kit (Hua Yue Yang Bio Co., Ltd., Beijing, China) according to the manufacturer’s instructions, and the genomic DNA residues were removed during RNA extraction. RNA concentration and quality were tested in an Agilent 2100 Bioanalyzer (Agilent, Santa Clara, CA, USA). RNA quality was also confirmed by RNase free agarose gel electrophoresis. RNA-seq libraries were constructed as previously described [61] and sequenced on the Illumina HiSeq™ 2000 platform (Illumina Inc., San Diego, CA, USA). Before assembly, adapter sequences were removed from the raw reads. Then low quality reads with over 50% bases with quality scores of 5 or lower and/or over 10% bases unknown (N bases) were removed from each dataset to gain more reliable results. After that, the clean reads of high quality from all the 18 samples were mapped to the longan genome databases [38], respectively. After alignments, raw counts for each *D. longan* transcript and each sample were derived and were normalized to Reads Per Kilobase of transcript per Million mapped reads (FPKM). Differentially expressed genes (fold changes > 2 and adjusted *p*-value < 0.05) were identified by the DESeq package. The RNA-seq data have been uploaded to the NCBI Sequence Read Archive (SRS2241241, SRS2241242, SRS2241243, SRS2241244, SRS2241245, SRS2241246, SRS2241247, SRS2241248, SRS2241249, SRS2241250, SRS2241251, SRS2241252, SRS2241253, SRS2241254, SRS2241255, SRS2241256, SRS2241257 and SRS2241258).

### 4.5. Hormonal and Stress Treatments and Expression Profiling Using qRT-PCR

In this study, 27 one-year-old uniform grafted seedlings of “SJ”, obtained from the South Subtropical Crops Research Institute of the Chinese Academy of Tropical Agricultural Science in Zhanjiang (110°16′ E, 21°10′ N), were used for stress and hormonal treatments. For hormone treatments, three seedlings were treated with methyl jasmonate (MeJA) or SA solution (100 μM) for 4 h at 28 °C, respectively. Meanwhile, three seedlings sprayed with water were used as a control. For heat and cold stresses, three samples were grown at 42 °C or 0 °C for 4 h, respectively, and three samples grown at 28 °C were used as a control. All of the treatments were performed in a greenhouse. Six leaves were collected from each seedling and all samples were immediately frozen in liquid nitrogen and stored at −80 °C for expression analysis. Total RNA was extracted from leaves using the SuperFast new plants of RNA extraction kit while eliminate genome DNA following the manufacturer’s instructions (Hua Yue Yang Bio Co., Ltd., Beijing, China). First-strand cDNA was synthesized by reverse transcription of total RNA (500 ng) using PrimeScript RTase (TaKaRa Biotechnology, Dalian, China). Gene-specific primers were designed according to the *DlUBC* gene sequences using Primer Premier 5.0 and checked using BLASTn in NCBI (Table S1). In addition, the longan *Actin1* gene (Dlo_028674) was used as an internal control for normalization of the expression data. Real-time PCR was performed using a Bio-Rad real-time thermal cycling system (LightCycler 480; Bio-Rad Laboratories, Inc., Hercules, CA, USA) and SYBR-green to assess the expression levels of the candidate *DlUBC* genes. Each reaction consisted of 10 μL of 2 × SYBR Premix Ex Taq II (Takara Bio), 40 ng cDNA, and 250 nM of each primer in a final volume of 20 μL. The following PCR conditions were used: 94 °C for 15 min, followed by 40 cycles of 95 °C for 15 s, 58–63 °C for 20 s, and 72 °C for 30 s. The relative mRNA levels of the genes were measured using the cycle threshold (Ct) 2^(−Δ*C*t)^ method. The analysis included cDNA from three biological samples for each tissue, and all the reactions were run in triplicate. In the comparative expression analysis of *DlUBC* gene expression, genes that were up- or down-regulated at least two-fold were considered differentially expressed.

### 4.6. Statistical Analysis

Data were analyzed using variance (ANOVA) and the means were compared by the *t* test at the 5% level using the SPSS 11.5 software package (SPSS, Chicago, IL, USA).

## 5. Conclusions

A total of 40 putative *DlUBC* genes were identified in the longan genome and were grouped into 15 groups based on a phylogenetic analysis. The gene structure, conserved motifs, *cis*-elements and expression profiling, which may be related to their biological functions, were systematically analyzed. In each group, the exon-intron junctions and sequence motifs were highly conserved. The expression patterns of the *DlUBC* genes in various tissues showed that these genes might have important functions in longan growth and development. Based on our previous transcriptome data, we also analyzed the expression patterns of 40 *DlUBC* genes in two longan species during the three flowering stages. The results show that all the 40 *DlUBC* genes were constitutively expressed in all the three flowering stages in the “SX” longan variety, while 11 *DlUBC* genes were differentially expressed in the “SJ” longan variety. In addition, the expression levels obtained by qRT-PCR for six *DlUBC* selected genes (*DlUBC11*, *16*, *19*, *20*, *30* and *31*) were similar to the results obtained from the RNA-seq data. The expression results suggest that *DlUBC* genes may be involved in the regulation of flower induction. Furthermore, the expression patterns of *DlUBC* genes show that they play potentially important roles in mediating the effects of stress induced by SA, MeJA, and extreme temperatures. The results of our study establish a foundation for future studies on the functions of *DlUBC* genes in organ development and plant stress response, and for further elucidation of the potential functions of the *DlUBC* genes in longan varieties.

## Figures and Tables

**Table 1:** The information of *DlUBC* gene family.

Gene Name	Gene Locus ID	Location	ORF (bp)	Size (aa)	UBCc Domain	PI	MW (KDa)	Intron	Full Length
*DlUBC1*	Dlo_000106.1	scaffold1:985697:987091	567	188	32–174	7.08	21.65	4	1395
*DlUBC2*	Dlo_000292.1	scaffold1:3030633:3033521	483	160	8–158	8.42	18	4	2888
*DlUBC3*	Dlo_026265.1	scaffold6:2172282:2176388	459	152	7–150	5.37	17.35	4	4106
*DlUBC4*	Dlo_032644.1	scaffold9:121161:124354	519	172	29–172	6.82	18.74	3	3193
*DlUBC5*	Dlo_002066.1	scaffold11:28768:34251	447	148	4–147	7.72	16.49	3	5483
*DlUBC6*	Dlo_009607.1	scaffold19:759717:760407	330	109	1–107	6.50	12.75	2	691
*DlUBC7*	Dlo_011099.1	scaffold21:38074:42650	2769	922	676–833	4.77	102.86	6	4576
*DlUBC8*	Dlo_012344.1	scaffold23:232655:235481	528	175	1–139	4.18	19.99	5	2826
*DlUBC9*	Dlo_014547.1	scaffold27:1628072:1630343	462	153	8–152	4.83	17.48	3	2272
*DlUBC10*	Dlo_016672.1	scaffold32:473094:475341	537	178	1–139	4.16	20.08	5	2247
*DlUBC11*	Dlo_021485.1	scaffold45:1169893:1172427	486	161	18–161	7.00	18.43	5	2534
*DlUBC12*	Dlo_024241.1	scaffold54:747570:752709	3438	1145	894–1054	4.57	126.46	6	5139
*DlUBC13*	dlo_034965.1	scaffold54:903777:904902	447	148	4–147	7.68	16.51	3	1125
*DlUBC14*	Dlo_024956.1	scaffold56:49487:50629	1143	380	79–236	9.82	42.60	0	1142
*DlUBC15*	Dlo_024957.1	scaffold56:57967:58685	582	193	1–136	9.08	21.98	1	719
*DlUBC16*	Dlo_024978.1	scaffold56:225690:227300	915	304	14–174	5.89	34.00	4	1610
*DlUBC17*	Dlo_024979.1	scaffold56:228547:229700	540	179	14–155	6.59	20.69	3	1153
*DlUBC18*	Dlo_026358.1	scaffold60:843621:845935	447	148	4–147	7.69	16.39	3	2314
*DlUBC19*	Dlo_032160.2	scaffold87:1001138:1002555	612	203	61–203	5.50	22.39	6	1417
*DlUBC20*	Dlo_033394.1	scaffold94:238050:239736	330	109	1–107	5.50	12.44	3	1686
*DlUBC21*	Dlo_034066.1	scaffold99:423532:424482	267	88	1–86	8.82	9.90	4	950
*DlUBC22*	Dlo_001351.1	scaffold105:47204:51064	447	148	4–147	7.72	16.58	3	4250
*DlUBC23*	Dlo_005400.1	scaffold139:303181:306533	585	194	5–150	4.72	21.26	4	3352
*DlUBC24*	Dlo_009107.1	scaffold182:25126:28034	630	209	74–173	5.30	24.28	4	2908
*DlUBC25*	Dlo_009840.1	scaffold192:53161:56243	447	148	4–147	7.72	16.55	3	3082
*DlUBC26*	Dlo_009841.1	scaffold192:56889:58714	474	157	2–131	8.44	17.85	3	1825
*DlUBC27*	Dlo_010855.1	scaffold204:275311:285221	2088	695	332–489	4.79	77.60	7	9890
*DlUBC28*	Dlo_013399.1	scaffold247:138383:139816	447	148	4–147	7.72	16.46	3	1433
*DlUBC29*	Dlo_013592.1	scaffold250:137505:139550	552	183	32–174	8.61	21.01	4	2045
*DlUBC30*	Dlo_015182.2	scaffold286:460274:460720	447	148	29–142	8.55	17.22	0	446
*DlUBC31*	Dlo_016190.1	scaffold303:303486:307073	1617	538	258–415	5.99	59.98	6	3587
*DlUBC32*	Dlo_017847.1	scaffold347:238580:242477	735	244	1–132	9.60	26.47	4	3897
*DlUBC33*	Dlo_019295.1	scaffold388:24595:27323	1731	576	9–161	8.39	63.39	5	2728
*DlUBC34*	Dlo_021725.2	scaffold459:127808:133234	462	153	8–151	6.74	17.21	7	5426
*DlUBC35*	Dlo_031542.1	scaffold832:7085:11030	717	238	8–162	8.78	26.98	8	3945
*DlUBC36*	Dlo_032039.1	scaffold860:46363:48204	315	104	1–89	4.63	11.71	2	1841
*DlUBC37*	Dlo_001660.1	scaffold1077:94570:95523	954	317	68–228	6.08	37.35	0	953
*DlUBC38*	Dlo_006580.1	scaffold1489:71154:72782	483	160	8–158	8.39	18.06	4	1628
*DlUBC39*	Dlo_008135.1	scaffold1681:29423:31993	510	169	4–134	4.33	19.25	4	2570
*DlUBC40*	Dlo_008607.1	scaffold17588:7:484	243	80	2–68	4.12	9.04	2	478

## Supplementary Materials

The following are available online. Table S1: Primers used in quantitative RT-PCR of *DlUBC* genes. Table S2: The candidate UBC genes and their protein structure found in longan genome. The different colors indicated the redundant genes. Table S3: The information for each motif of *DlUBCs*. Table S4: FPKM values of *DlUBC* genes in nine tissues of longan. Table S5: FPKM values of *DlUBC* genes in the three flowering stages of “Sijimi” (SJ) and “Shixia” (SX) longan species. Red color indicates the genes which showed down-regulated expression; blue color indicates the genes which showed up-regulated expression; and green color indicates the genes showed an up-regulated expression in the first two stages and a down-regulated expression in the third stage. Table S6: Details of the cis-elements identified in this study. Table S7: Predicted cis-elements in the promoter of the *DlUBC* genes. Figure S1: Conserved motifs of *DlUBC* proteins. (a) The neighbor-joining (NJ) tree on the left includes 40 *DlUBC* proteins from longan. Different colors represent various groups; (b) The conserved motifs of *DlUBC* proteins. MEME was used to predict motifs, and these motifs are represented with boxes.
